# Microstructure, Compressive Strength and Sound Insulation Property of Fly Ash-Based Geopolymeric Foams with Silica Fume as Foaming Agent

**DOI:** 10.3390/ma13143215

**Published:** 2020-07-19

**Authors:** Xinhui Liu, Chunfeng Hu, Longsheng Chu

**Affiliations:** School of Materials Science and Engineering, Southwest Jiaotong University, Chengdu 610031, China; XinhuiLiu@my.swjtu.edu.cn (X.L.); Chfhu@live.cn (C.H.)

**Keywords:** geopolymeric foam, microstructure, compressive strength, sound insulation performance

## Abstract

Geopolymer as an alternative to cement has gained increasing attention. The aim of this article is to study the influence of the silica fume content and activator type on the porous fly ash-based geopolymer with silica fume as foaming agent. Geopolymeric foams were fabricated using low-calcium fly ash, silica fume, and sodium-based alkaline activator as initial materials. The designed silica fume contents were 0, 15, 30, and 45 wt % and two kinds of activators of water glass and sodium hydroxide were used for comparison. Phase composition, microstructure, mechanical properties and sound insulation properties of as-prepared bulks were systematically investigated. It was found that, with increasing silica fume content, the density and compressive strength decreased simultaneously, whereas the porosity and sound insulation performance were effectively enhanced. At the silica fume content of 45% with sodium hydroxide as activator, the porosity was increased 3.02 times, and, at the silica fume content of 45% with water glass as activator, the mean sound insulation value of 43.74 dB was obtained.

## 1. Introduction

Geopolymers have been gained increasing attention since the 1990s owing to their light weight and low energy consumption. To date, the known reaction mechanisms of geopolymerization process was the theory of depolymerization and polycondensation proposed by Davidovits, in which the silicon (Si)–oxygen and aluminum (Al)–oxygen bonds are broken and recombined to form a three-dimensional polymer network under the motivation of an alkaline activator [1]. Although the specific reactions are not the same for different alkaline activators and raw materials, the backbone reactions are similar. The basic structure in the alkali-activated system is a highly crosslinked and disordered aluminosilicate gel. Both Si and Al exist in tetrahedral coordination, and the tetrahedral charge balance is achieved by the association of the alkali metal cation with the gel skeleton [1].

It has been reported that the lightweight geopolymers possess excellent properties of low shrinkage [2], high sound absorption, high thermal insulation [3,4,5], and high resistance to acids [6]. Generally, the lightweight geopolymers are fabricated by foaming process. Physical and chemical foaming are two common foaming ways, in which the physical foaming is associated with the foaming agents of detergents, glue resins, saponin, resin soap, and hydrolyzed proteins. A large quantity of bubbles is formed in situ [7,8]. Chemical foaming is a process of reaction among hydrogen peroxide, sodium hypochlorite, Al, Si, and alkaline solution to generate gas to form pores in the geopolymer [9]. In previous works, Szabó et al. obtained a fly ash (FA)-based geopolymer via foaming with hydrogen peroxide, yielding a low-density (0.52 g/cm^3^) with a high compressive strength of 0.9 MPa [10]. Feng et al. used hydrogen peroxide as the blowing agent to obtain a FA-based geopolymeric foam with 79.9% porosity, 0.0744 W/(m·K) thermal conductivity, and 0.82 MPa compressive strength [11]. Böke et al. used sodium hypochlorite as the foaming agent to obtain geopolymeric foam with 55% porosity, presenting the compressive strength of 3.10 ± 18% MPa [12]. In addition, Ducman et al. obtained a low-density (0.64–0.74 g/cm^3^) FA-based geopolymeric foam with a high compressive strength (3.3–4.3 MPa) by adding 0.07–0.2% aluminum fume as the pore generation agent [13].

Additionally, noise is considered to be one of the serious environmental pollutions in daily life. It generally comes from transportation, production, and living noise. Exposure to noise can lead to many negative effects, such as lack of comfort, health problems, reduced privacy, and sleep disturbances [14]. These adverse effects make it particularly important to isolate the propagation of sound from one space to another through buildings. When silica fume (SF) is used as the foaming agent during reactions with various clays (kaolin, metakaolin, illite, and montmorillonite), an inorganic foam is produced via a rapid, low-energy reaction [15]. However, there are few studies on the effect of SF as foaming agent on the FA-based geopolymeric foams. FA and SF are industrial waste, and their open storage will occupy the land and cause dust pollution. Using them as a substitute for cement also can reduce the electricity and heat energy used in the process of cement production [16]. In the previous exploration, it was found that SF will generate connected pore structures in the geopolymerization reaction of fly ash. The generation of open pore structure provides a possibility to obtain a lightweight geopolymer material with good sound insulation properties. In this paper, the influence of silica fume content and activator type on the porous fly ash-based geopolymer with silica fume as foaming agent were studied. Two kinds of alkaline activators were selected to fabricate FA-based geopolymeric foams. The microstructure, compressive strength, and acoustical performance were systematically investigated.

## 2. Materials and Methods

### 2.1. Materials

SF and FA were used as initial materials to prepare geopolymeric foams. FA was a low calcium byproduct coming from burning pulverized coal in a coal combustion power plant (Gongyi, China). The primary phases in FA were quartz and mullite. The specific gravity of FA was 2.11 g/cm^3^. The particle size was 43 μm and the specific surface area was 360 m^2^/kg. SF was obtained by collecting dust when smelting ferrosilicon alloy or metal silicon. The condensation process was very rapid, and SiO_2_ was too late to generate crystalline substances. The existence of a large amount of amorphous phase makes SF possess great pozzolanic activity. The specific gravity of SF was 1.80 g/cm^3^. The particle size was 0.15 μm and the specific surface area was 20,000 m^2^/kg. The chemical compositions of the raw materials are shown in Table 1. Sodium hydroxide pellets (98.0% purity) and water glass were used to produce the alkaline activators. The molar concentration of the sodium hydroxide solution is 3 mol/L (pH = 14), and the modulus of the water glass (NaO_2_∙nSiO_2_) is 1.5 (*n* = 1.5, pH = 14). Both the activator solutions were aged for one day before using. 

### 2.2. Methods

The proportioning designs of the FA-based geopolymeric foams are given in Table 2. Firstly, FA and SF were mixed using a cement paste mixer (Huanan Laboratory Apparatus, Wuxi, China) for 1 min (700 r/min) and then the activator solution was added and the slurry was mixed for 5 min (1400 r/min). The obtained pastes were poured into cylindrical (100 mm diameter) and square metallic molds (40 mm × 40 mm × 160 mm), and subsequently covered with polyethylene films. Then the samples were cured for 24 h at 80 °C followed by placing at ambient temperature for 27 days. The cylindrical samples were cut into uniform cylinders with a thickness of 28 mm for sound insulation performance testing.

The phase composition of the geopolymers was examined using a Empyrean diffractometer (PANalytical, Netherlands) with a copper target at 40 kV and 40 mA. The scanned range was 5–85° and the scanning speed was 0.013 s/step. The microstructure morphology of samples was observed using a QUANTA 200 SEM (Thermo Scientific, Waltham, MA, USA) at a voltage of 20 kV. To get good electrical conductivity, all samples were coated with gold. The compressive strength of specimens were measured using an intelligent manometer (RFP-03 Intelligent Pressure Meter; Qiaoke, Hebei, China) according to the standard GB50081-2002 [17]. At least three samples were used for each measurement. 

The measurement method of the volume density of geopolymer is as follows:

1. Place the sample in an oven to dry to constant weight. The mass of the sample is recorded for m_0_.
2. Immerse the sample in distilled water after cooled to room temperature. Remove the sample after 48 h and wipe the surface with a wet towel wringed out the water. The mass of the sample is weighed and recorded it as m_1_.
3. Weigh the water-saturated sample in water and record it as m_2_.

ρ_b_ =ρ_w_ × m_0_/(m_1_ − m_2_) (1)

where ρ_b_ is the volume density of the sample (g/cm^3^); ρ_w_ is the density of water at room temperature (g/cm^3^); m_0_ is the mass of the dried sample in air (g); m_1_ is the mass of water-saturated sample in the air (g); m_2_ is the mass of water-saturated sample in water (g).

The porosity refers to the percentage of the volume of pores in the bulk material to the total volume of the material in its natural state. The measurement method of the porosity of geopolymer is as follows:

1. Grind the sample into powder and place the powder in an oven to dry to constant weight.
2. Weigh the powder with a mass of 10 g and record it as m’_0_, then put the sample into the cleaned pycnometer.
3. Add distilled water to the pycnometer to the mark, weigh its mass, and record it as m’_2_.
4. Add distilled water to the clean pycnometer to the mark, weigh its mass, and record it as m’_1_.

ρ_t_ =ρ_w_ × m’_0_/(m’_1_ + m’_0_ − m’_2_) (2)

where ρ_t_ is the true density of the sample (g/cm^3^); ρ_w_ is the density of water at room temperature (g/cm^3^); m’_0_ is the mass of dry powder sample in the air (g); m’_1_ is the mass of the pycnometer only containing distilled water (g); m’_2_ is the mass of the pycnometer with powder and water (g).

ρ_a_ = (1 − ρ_b_/ρ_t_) × 100% (3)

where ρ_a_ is the porosity of the sample; ρ_b_ is the volume density of the sample (g/cm^3^); ρ_t_ is the true density of the sample (g/cm^3^).

The sound insulation performance was tested by a VA-Lab four-channel test system (BSWA TECH, Beijing, China), which consisted of two different impedance tubes: a 100 mm diameter tube (SW422) for the 63–1600 Hz testing and a 30 mm diameter tube (SW477) for the 1000–6300 Hz testing. 

The sound insulation performance of materials is related to the frequency. According to GB/T 1988 9.3–2005 [18], the laboratory generally adopts one-third octaves, and the center frequencies of 100, 125, 160, 200, 250, 315, 400, 500, 630, 800, 1000, 1250, 1600, 2000, 2500 and 3150 Hz are measured. In order to express simplicity and facilitate comparison, the mean sound insulation value is often used as the evaluation standard in engineering. The calculation formula is as follows:

(4) $$\bar{R}=\frac{R_{1}+{\;R}_{2}+\cdots +{\;R}_{i}}{n}$$

where $\bar{R}$ is the mean sound insulation value (dB), R_i_ is the value of sound insulation of a given one-third octave band, *n* is the number of sound insulation bands (*n* = 16).

## 3. Results

### 3.1. Composition and Microstructure Analysis

**Figure 1:**
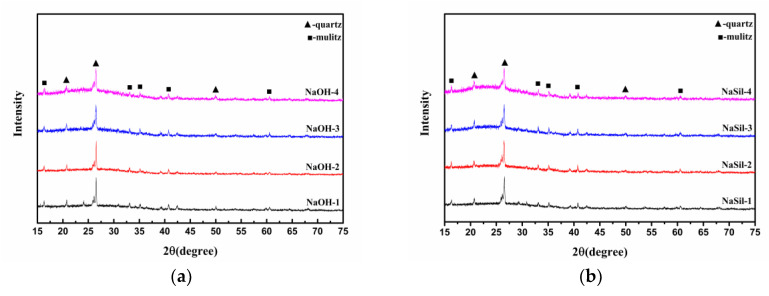
X-ray diffraction (XRD) spectra of the geopolymers fabricated using (**a**) sodium hydroxide activating solution and (**b**) water-glass activating solution.

The compositions of as-prepared FA-based geopolymeric foams were examined and are shown in Figure 1. The horizontal axis shows X-ray diffraction (XRD) angle (2θ), and the vertical axis shows diffraction intensity. It is seen that the phase compositions of FA-NaOH and FA-NaSil are similar, with the existence of primarily amorphous phase, quartz, and mullite. The diffraction peaks at 2θ = 20–40° are both diffuse and taro-shaped, and represent primarily amorphous aluminosilicate gel phases [19]. The diffraction peaks of aluminosilicate gel phase are shifted to the right relative to the amorphous phases of SF and FA, implying that “depolymerization-rearrangement” process occurred during the chemical treatment [20,21]. By increasing the SF content and changing the alkaline activator, the compositions of FA-based porous materials did not change. Additionally, it is observed that, with the increment of SF content, the peaks of the quartz and mullite decrease and the peak area of the taro-shaped peak increases, indicating that more crystals were consumed and more amorphous phase appeared. Furthermore, the XRD peak between 20° and 40° is higher for the water-glass activating solution than that for the hydroxide activating solution. Although both activating solutions have a pH of 14, the hydroxide activating solution yields a lower reactivity than the silicate activating solution during the geopolymerization reaction [22].

**Figure 2:**
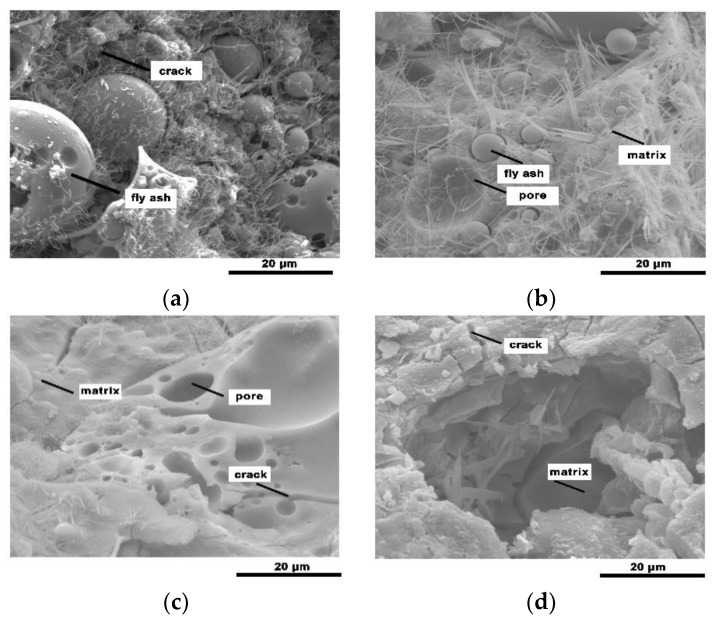
Scanning electron microscope (SEM) images of the obtained geopolymers: (**a**) NaOH-1; (**b**) NaOH-2; (**c**) NaOH-3; (**d**) NaOH-4.

**Figure 3:**
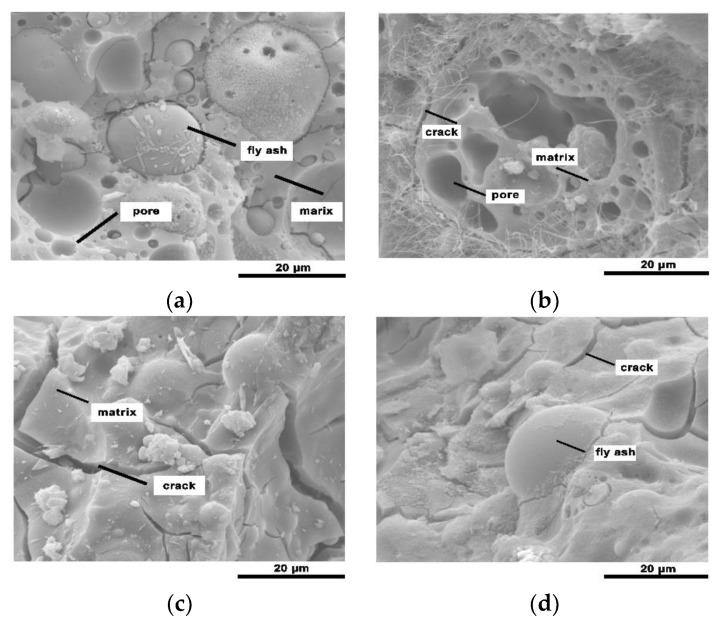
Scanning electron microscope (SEM) images of the obtained geopolymers: (**a**) NaSil-1; (**b**) NaSil-2; (**c**) NaSil-3; (**d**) NaSil-4.

The geopolymeric foam microstructures of Figure 2 and Figure 3 show the reaction degree of the geopolymerization reaction. FA is composed of hollow vitreous spheres of varying sizes, some of which contain other smaller spheres [23]. Mineral particles melt to form small spherical droplets during the coal combustion process via sudden cooling and surface tension [24]. The unreacted FA particles in SEM images illustrate the general FA structure. Large and small honeycomb holes, and unreacted FA particles are observed on the FA-based geopolymer surface, and the FA particles are not completely covered by the aluminosilicate gel with clear gaps between the particles and gel products (Figure 2a and Figure 3a). The floccule on the surface of FA particles indicates that the FA particles gradually react from the particle surface to the interior during the geopolymerization reaction. The quantity of unreacted FA particles on the surface of the geopolymeric foams decreases as the SF content increases, and most of the FA particles are covered by the aluminosilicate gel which fills the gaps between the FA particles and gel products. 

The water-glass-activated FA-based geopolymeric foams underwent a more complete reaction than the sodium-hydroxide-activated foams. Some unreacted FA particles are embedded in the homogeneous amorphous matrix with the bulk of the smaller FA particles potentially dissolving and forming the aluminosilicate gel (Figure 3a), whereas lots of small FA particles are clearly visible in Figure 2a. The hydroxide-activated geopolymerization reaction is slower than the silicate-activated geopolymerization reaction due to the increasing Na/Si ratio [25]. And Figure 2 shows more needle crystals than Figure 3, which is a product formed by the combination of incompletely reacted alkaline activator and water and might have a negative impact on the compressive strength of the geopolymeric foams. Furthermore, cracks are observed in the SEM images of the geopolymeric foams. The size of the cracks is 0.07–2.5 μm, and the mean size is 0.5 μm. There is no obvious difference in the size of the cracks of different alkaline activators. The existence of cracks reduces the compressive strength by increasing water absorption and generating through-holes in the geopolymers. On the other hand, more through-holes may have a beneficial effect on sound insulation performance.

### 3.2. Physical and Mechanical Properties

**Figure 4:**
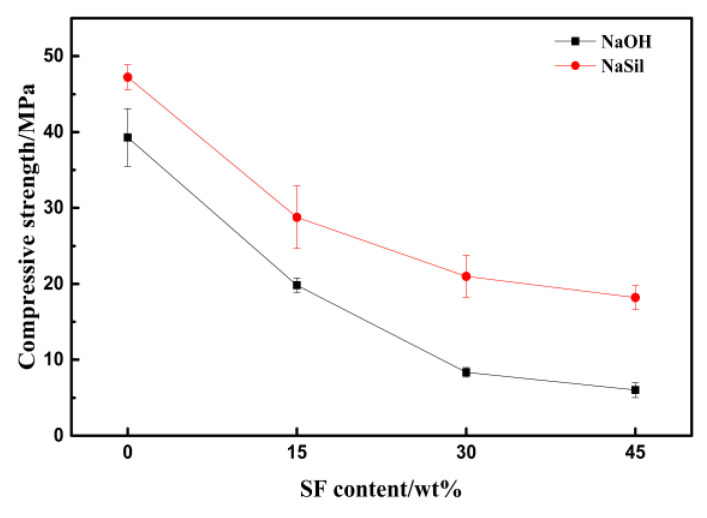
Compressive strength of geopolymers function of SF content.

In Figure 4, as the content of SF increases, the compressive strength of the geopolymeric foam decreases, but the downward trend is slowing down. The geopolymeric foam fabricated using water glass as the alkaline activator has a higher compressive strength. When the SF content increases, the compressive strength drops slower than that with the sodium hydroxide solution as the alkaline activator. Luna-Galiano et al. noted that the hydroxide-activated geopolymerization reaction had a lower reactivity than the silicate-activated reaction, yielding a lower geopolymeric gel content in the microstructure, a more porous structure, and poor mechanical performance [25].

**Figure 5:**
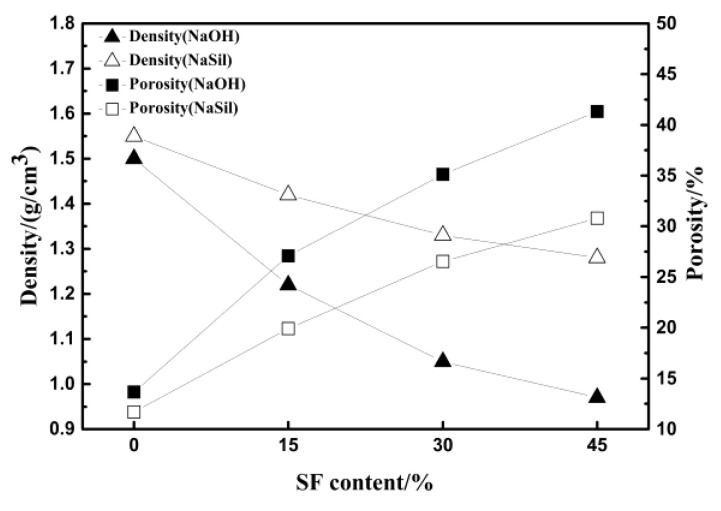
Density and porosity of geopolymers versus SF content.

In Figure 5, the overall trend of density and porosity is opposite. Increased porosity makes the percentage of the volume of pores in the geopolymer improved, that is, the density is reduced. The increase of porosity decreases the compressive strength. For the porous geopolymers, the gelling matrix is the main load-bearing site of the force. The increase of porosity reduces the load-bearing site of force per unit area, and the densely distributed pores make the cracks easier to propagate. The compressive strength decreased by 84.70%, about a 3.02 times increase in the porosity as the SF content increased from 0 to 45% when the sodium hydroxide was used as the activator. Whereas the compressive strength decreased by 61.41% and there was a 2.63 times increase in the porosity when the water glass was used as the activator. The use of sodium hydroxide as the alkaline activator could therefore yield lighter and more porous geopolymers, and the water glass as the alkaline activator could result in geopolymeric foam with higher compressive strength.

**Figure 6:**
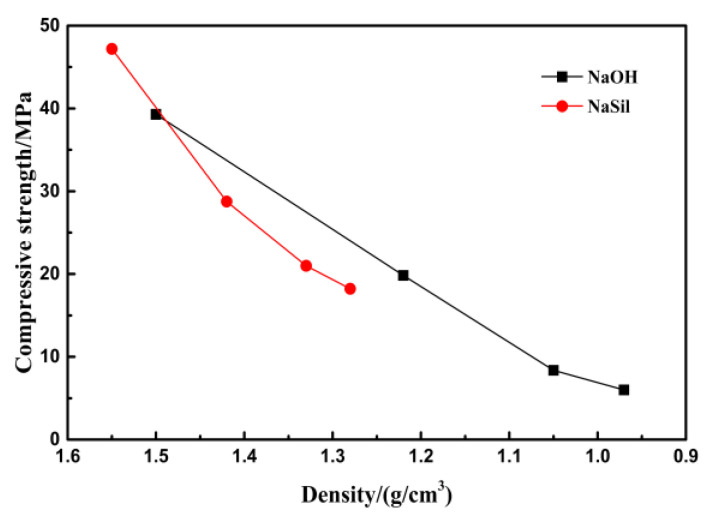
Compressive strength of geopolymers function of the density.

The compressive strength of foamed cement concrete decreases exponentially as the density decreases generally. It can be seen in Figure 6 when the sodium silicate is used as an activator the downward trend of compressive strength is slower with the decrease of density. When the sodium hydroxide is used as the activator and the silica fume content is increased to 45%, the downward trend of the compressive strength is slower with the decrease of density, too. The addition of silica fume makes the gel matrix denser, and the pore wall has a good supporting effect. It can be concluded that, compared to other foaming methods, the silica fume as a pore-forming agent might be more conducive to obtaining high-strength porous materials.

### 3.3. Sound Insulation Performance

**Figure 7:**
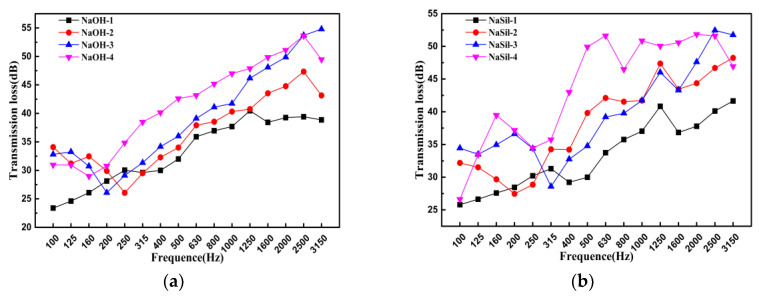
Sound transmission loss of the geopolymers fabricated using a (**a**) sodium hydroxide activating solution and (**b**) water glass alkaline activating solution.

Figure 7 presents the experimental curves of the sound transmission loss (STL) as frequency for geopolymeric foams in this study. As the frequency increases, the overall STL shows an upward trend. Figure 7a shows that when the frequency is higher than 315 Hz, the proportions of SF are greater in the mixture, and the STL is higher. In the case of adding SF, transmission loss troughs are formed in 250 Hz, 200 Hz and 160 Hz, respectively. It indicates that the coincidence effect has occurred. In Figure 7b, the STL of geopolymeric foam has a large fluctuation with increasing frequency, but it still can be seen that the sound insulation effect is the best when the SF content is 45%. By calculating the mean sound insulation value, the proportions of SF are greater in the mixture, and the mean sound insulation value is better (Table 3). In addition, the sound insulation performance of geopolymeric foam using the water glass as activator is better than that with the sodium hydroxide as activator. When the SF content is 45% with the water glass as activator, the mean sound insulation value is maximum.

## 4. Discussion

### 4.1. Composition and Microstructure Analysis

The geopolymeric foam preparation process can be described as three stages: bubble formation, viscosity increase, and material consolidation. Two reactions occur during the process: the geopolymerization reaction and the pore generation reaction [25]. Here the geopolymerization reaction occurs when the FA reacts with either sodium hydroxide or the water glass to form aluminosilicate gel. The pore generation reaction is based on inorganic in situ foam formation theory. The pores might be caused by the hydrogen produced during the reduction reaction of water (Equation (5)), silicon oxidation (Equation (6)), and the formation of orthosilicic acid species (Equation (7)) [26].

4H_2_O + 4e^−^ → 2H_2_ + 4OH^−^ (5)

Si^0^ → Si^4+^ + 4e^−^ (6)

4H_2_O + Si^0^ → 2H_2_ + Si(OH)_4_ (7)

**Figure 8:**
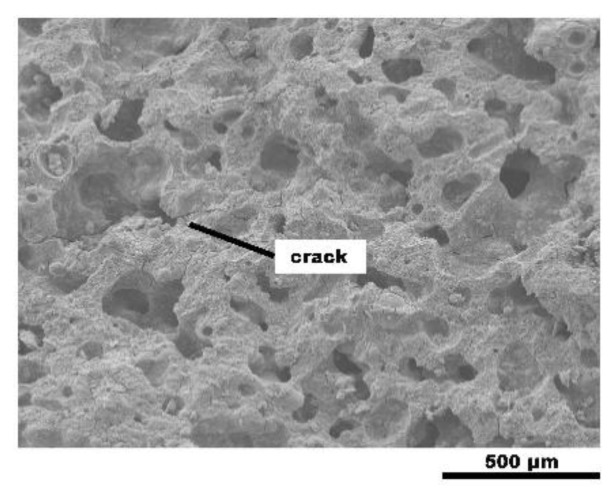
Scanning electron microscope (SEM) images of the obtained geopolymeric foam.

This is because the material consolidation process is slow and the gas produced by pore generation reaction has a tendency to be expelled outward. Moreover, the additive of SF increases the fluidity of the geopolymer slurry, and reduces the viscosity. Therefore, the resistance during the movement of bubbles reduces. The movement paths of bubbles cross and generate interconnected pores in the geopolymer matrix (Figure 8).

The addition of SF significantly improves the reactivity of FA. The viscosity of the FA-based geopolymer slurry is high, which reduces the fluidity of the slurry so that the FA particles cannot react completely. The addition of SF will introduce a mass of bubbles that increases the fluidity of the slurry and allows the FA particles to react more completely. Moreover, SF can increase the amount of hydration product. The SF hydration reaction produces C–S–H gel, and the C–S–H gel simultaneously provides a nucleation site for the formation of the silicoaluminate gel. Therefore, the increasing content of SF promotes geopolymerization. But quartz and mullite are inert in the geopolymerization reaction and cannot fully participate in the reaction [27]. When the content of SF reaches 45%, the amount of quartz and mullite in the system is still very considerable. In addition, although the quantity of the alkaline activator in this experiment does not change, the quantity of the alkaline activator reacting with the FA per unit mass increases as the replacement ratio of the SF increases. The increased alkali content also promotes the geopolymerization. More gel formation makes the pore walls of the geopolymeric foam more continuous and denser.

### 4.2. Physical and Mechanical Properties

The particle size of SF is much smaller than that of FA. The addition of SF makes particle gradation more reasonable of the slurry, and the fine SF particles will play a lubricating role of increasing the fluidity of the slurry and reducing the mixing water. The FA particles are surrounded by water. The surface tension generated on the surface of the water film will cause a gap around the FA particles. After the depolymerization and polycondensation reaction of the FA, the inside of the gel products will become loose. SF particles are relatively small and will be distributed around the FA particles, making the slurry particle distribution more reasonable and the gel product denser. In addition, because the specific surface area of SF particles is relatively large, in order to reduce the surface energy, SF particles will adsorb water molecules on the surface for reducing the probability of bleeding. When SF content increases, the amount of alkali reacting with FA is also relatively increased. When the alkali content increases, the rate of polycondensation and hydration is accelerated, increasing the amount of soluble A1 in the initial stage of the reaction. At the same time, in the high alkaline conditions, the solution phase is mainly composed of monomer [SiO_4_] and smaller oligomer chain, which are prone to polymerization with soluble Al to form a binder phase. Furthermore, SF-hydrates form C-S-H gel. Under the combined effect of the increase of gel products and the micro-aggregate effect of SF, as well as the pore formation reaction, the tendency of compressive strength and density decrease, and the porosity increases slow down

### 4.3. Sound Insulation Performance

When there are open pores in the material, sound waves transmit through the connected small holes of the material. Due to the viscous resistance of the air, the air in the hole and the wall of the hole will have a friction effect. At the same time, when the air in the small hole is compressed, the temperature in the hole increases; when the air is sparse, the temperature in the hole decreases. By heat conduction, part of the sound energy is converted into heat energy and lost in the environment. The absorption of sound energy is increased, and the sound insulation performance of the material is enhanced. The increase of SF content makes more hydrogen generated and increases the fluidity of the slurry. The bubbles generated by foaming are difficult to be bound, they overlap each other to form more open pores.

In addition, as the content of SF increases, the reactivity of geopolymerization increases. When there are many FA particles in the slurry that has not reacted, the gel product cannot form a dense entity. The addition of SF increases more; the pore walls are denser. Although increasing the content of SF, a large number of pores overlap, leading to an increase in poor pore structure, but the increased gel products make up for this defect. The dense matrix is beneficial to increase the reflected sound energy of the material and improve the sound insulation.

The dense hole wall is more conducive to sound insulation. Using the sodium hydroxide as activator causes a higher porosity, but the degree of geopolymerization reaction is lower than that of water glass as activator; the hole wall is less dense.

## 5. Conclusions

The microstructure, compressive strength, and sound insulation performance of fly ash-based geopolymers were systematically investigated with SF content and activator type as variables. With the increasing SF content, the porosity increased relative to the decreasing density and compressive strength. When using the water glass as the activator, the density and compressive strength had the higher values than that of foams using the sodium hydroxide as the activator. At the SF content was 45% with the sodium hydroxide as activator, the porosity of geopolymeric foam could be increased for 3.02 times. Regardless of sodium hydroxide or water glass as the activator, the sound insulation performance of the geopolymeric foam was improved with the increase of SF content. Although using the sodium hydroxide as activator could make geopolymeric foam have higher porosity, using the water glass as an activator induced better sound insulation performance. When the SF content was 45% and the water glass was used as the alkaline activator, the mean sound insulation value was maximum.

Silica fume and fly ash are industrial wastes, and their prices are lower than that of cement. Using them as initial materials to fabricate geopolymeric foams not only brings environmental benefits, but also brings economic benefits. The geopolymeric foam is expected to be used as no-load-bearing walls. Because of its characteristic of lightweight, it will have great advantages for prefabricated buildings especially. It can reduce the transportation and installation costs. 

## Figures and Tables

**Table 1:** The chemical composition of fly ash (FA) and silica fume (SF).

Raw Materials	Mass Percent/wt %						
	SiO_2_	Al_2_O_3_	Fe_2_O_3_	CaO	MgO	K_2_O	Na_2_O
FA	51.44	31.48	5.59	5.29	1.02	1.71	0.73
SF	93.35	2.96	0.14	0.63	0.75	0.59	-

**Table 2:** Proportioning design of the fly ash (FA)-based geopolymers.

Samples	Solid Phase (wt %)		Alkaline Activator (g)/Solid Phase (g)	
	FA	SF	NaOH	Water Glass
NaOH-1	100	0	0.37	-
NaOH-2	85	15	0.37	-
NaOH-3	70	30	0.37	-
NaOH-4	55	45	0.37	-
NaSil-1	100	0	-	0.37
NaSil-2	85	15	-	0.37
NaSil-3	70	30	-	0.37
NaSil-4	55	45	-	0.37

**Table 3:** The mean sound insulation value of geopolymers (dB).

**NaOH-1**	**NaOH-2**	**NaOH-3**	**NaOH-4**
33.18	36.62	39.26	41.56
**NaSil-1**	**NaSil-2**	**NaSil-3**	**NaSil-4**
33.31	38.34	39.48	43.74
